# Correlations between ultrasound, tomographic, and intraoperative measurements of the great saphenous vein used as an arterial graft

**DOI:** 10.1590/1677-5449.202201212

**Published:** 2023-05-15

**Authors:** Vinicius Adorno Gonçalves, Daniel Martins Vieira Zimmermann, Fábio Hüsemann Menezes

**Affiliations:** 1 Universidade Estadual de Campinas - UNICAMP, Faculdade de Ciências Médicas, Hospital de Clínicas, São Paulo, SP, Brasil.

**Keywords:** saphenous vein, peripheral arterial disease, ultrasound, tomography

## Abstract

**Background:**

The great saphenous vein is the major superficial vein of the lower limb, and also the most often used as arterial graft material for lower limb revascularization. Prior knowledge of the quality of the vein can guide choice of therapeutic strategy, avoiding surgery that is doomed to failure. Discrepancies between intraoperative findings of the quality of the great saphenous vein and imaging tests are also frequently observed.

**Objectives:**

To evaluate the diameter of the great saphenous vein using two imaging methods (Duplex Ultrasound and Computed Tomography) and the gold-standard (intraoperative direct measurement of the vein), comparing the results.

**Methods:**

Prospective, observational study of data obtained during routine medical procedures performed by the Vascular Surgery team.

**Results:**

41 patients were evaluated, with a 12-month follow-up. 27 (65.85%) were male and mean age was 65.37 years. 19 (46.34%) patients had femoropopliteal grafts and 22 (53.66%) had distal grafts. Preoperative saphenous vein internal diameters measured with the patient supine were on average 16.4% smaller on CT and 33.8% smaller on US than the external diameters measured after intraoperative hydrostatic dilatation. There were no statistical differences in measurements when sex, weight, and height were considered.

**Conclusions:**

Saphenous vein diameters were underestimated by preoperative US and CT scans when compared to intraoperative measurements. Thus, in patients undergoing graft planning for revascularization, the choice of conduit should take this data into consideration, so that use of the saphenous vein is not ruled out unnecessarily during planning.

## INTRODUCTION

The great saphenous vein (GSV), also known as the long or internal saphenous vein, is the major vein of the lower limb. It originates medially from the dorsal venous arch of the foot and drains into the femoral vein, close to the inguinal ligament.^1-4^ In addition to venous drainage of the lower limb, the GSV also has an important surgical role because of its applications as an arterial graft material. One example is in myocardial revascularization surgery.^5^ This large vessel is also the first choice for use in vascular bypass surgery for lower limb peripheral vascular disease.^6^

Using the GSV for vascular surgery offers several advantages: (1) it is autologous, and it is known that autologous veins remain the best grafts, because they achieve the best rates of functionality over the long term for treatment of vascular diseases;^5^ (2) it is long and is easy to manipulate and dissect;^3^ and (3) it has large diameter and wall thickness, enabling construction of many different grafts. Primarily, it is clear that it is no coincidence that the GSV has been used for arterial grafts for more than 50 years.^7^

Construction of arterial grafts using veins, in particular the GSV, involves detailed preoperative investigation. Assessment of the availability of the vein and of its quality is of great importance and should be performed before any surgical procedure. It can be achieved by physical examination and imaging exams, such as mapping with Duplex ultrasonography (US), computed tomography angiography (angio-CT), or magnetic resonance angiography.^1,5,8^ The quality and length of the vessel are assessed and veins with diameters from 2 to 3 mm should be evaluated (veins with diameters greater than 3 mm are considered good conduits), always seeking vessels that are malleable and compressible and rejecting any that are small, calcifed, or sclerotic.^5^ The diameter of the GSV is associated with its functionality as a graft and is therefore an important criterion, if not the most important, to define the appropriateness of using it as a substitute for an artery.^6^

While there are several studies that have correlated the different types of imaging exams with each other and with the functioning of the arterial graft constructed from the GSV, primarily with respect to the diameter of the vessel employed, no studies were found that report the correlation between the two types of examination and preparation of the vein during the surgical procedure.

## OBJECTIVES

### Primary objective

To assess GSV caliber comparing two imaging methods (duplex US and angio-CT) and a gold standard (intraoperative).

### Secondary objective

To determine whether age, sex, height, weight, or body mass index (BMI) affect intraoperative saphenous vein measurements.

## METHODS

A prospective, observational, non-interventional study in which the data analyzed were obtained during routine medical procedures performed by the vascular surgery team. The study was conducted within the Vascular Surgery Department, which is fully equipped to perform the imaging exams and surgical procedures. The project passed the Strengthening the Reporting of Observational Studies in Epidemiology (STROBE) checklist for cohort studies (available at: https://www.strobe-statement.org/checklists/) with analysis and validation of all 15 items on the list.

All patients signed free and informed consent forms and the study was approved by the institution’s Human Research Ethics Committee, protocol number 3.283.138, on April 25 of 2019; with Ethics Appraisal Submission Certificate (CAAE) number 10697019.1.0000.5404.

### Study population

The study population was elderly, comprising patients admitted to the vascular surgery ward for open revascularization of lower limbs using autologous GSV grafts. The decision to employ this treatment was taken during ambulatory consultations prior to admission of the patient and the imaging exams (US and angio-CT) were normally conducted in outpatients before admission. Data were collected on patients whose surgeries were conducted from September 2019 to December 2021. To avoid selection bias within a convenience sample, patients who underwent lower limb revascularization surgery with GSV grafts were listed for inclusion consecutively throughout the study period and all the data related to saphenous vein measurements taken using different methods were collected and compared for the same patient.

### Inclusion criteria

All patients admitted to the vascular surgery ward who underwent elective open lower limb revascularization surgery using autologous GSV grafts were invited to take part in the study.

### Exclusion criteria

Patients were excluded if they refused to participate in the study, did not sign the consent form, did not undergo revascularization surgery with a GSV graft, did not have angio-CT for preoperative planning, or did not have a US examination or intraoperative GSV caliber measurement and, therefore, the essential data needed for the study were not available.

The following variables were analyzed: age, sex, weight, height, and presence of chronic kidney disease, diabetes, arterial hypertension, and smoking. The distal site of graft anastomosis (whether to the popliteal artery or the distal arteries of the leg, such as the anterior tibial, posterior tibial, or fibular) and the rate of graft function while in hospital, at 30 days, and at 1 year were analyzed. Additionally, BMI was calculated and the GSV diameter was measured (with angio-CT, US, and during surgery) at the arch of the saphenous vein and 10 cm, 20 cm, 30 cm, 40 cm, 50 cm, and 60 cm below the arch.

### Assessment by angiotomography

**Figure 1:**
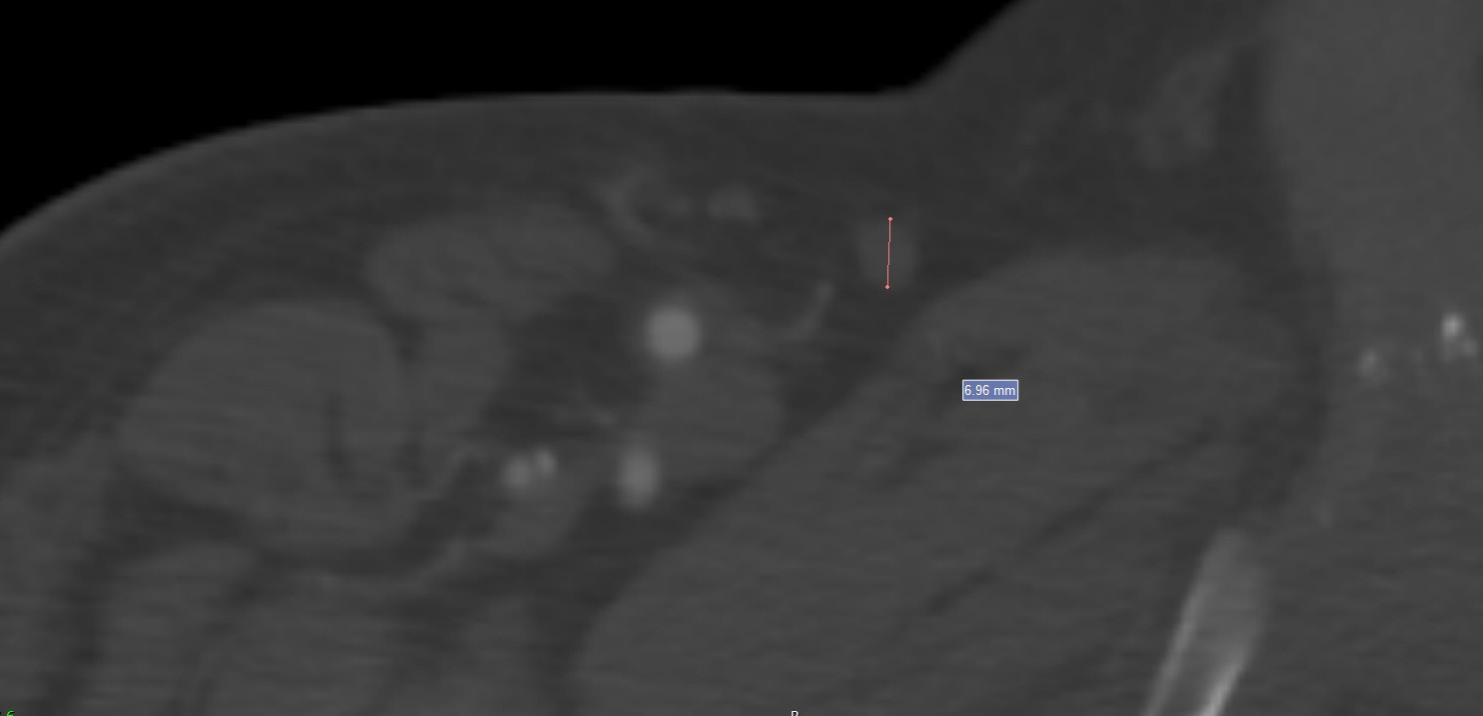
Measurement of great saphenous vein diameter with angiotomography.

Angio CT of the abdominal aorta and the lower limb arteries was conducted to assess the limb arterial bed for surgical planning using a Toshiba Medical Aquilion 64 machine. The examination was ordered by the medical team according to the radiology protocol. Saphenous vein measurements were taken by the same author (VAG) along the axis perpendicular to the skin, measuring the external diameter of the vessel wall (Figure 1).

### Ultrasonographic assessment

**Figure 2:**
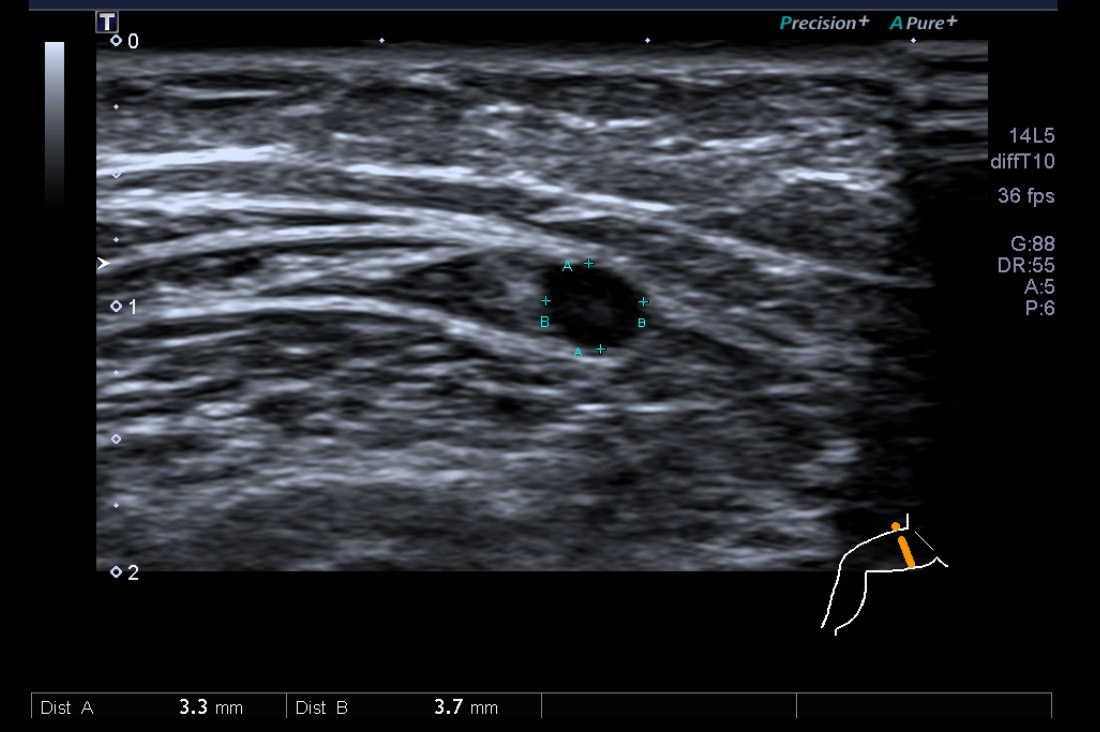
Measurement of great saphenous vein diameter with ultrasonography.

Patients scheduled for revascularization surgery were routinely scheduled for assessment of GSV caliber with US. This examination is noninvasive and painless and was conducted by an assistant physician from the vascular surgery team in the vascular laboratory noninvasive procedures room, or in the operating room immediately before anesthesia for the surgical procedure, under supervision by one of the authors (FHM), using a Toshiba Aplio 500 US machine. The saphenous vein measurements were taken along an axis perpendicular to the skin, taking the external diameter of the wall of the vessel at the point at which the image is circular, with minimal pressure exerted on the skin by the transducer (Figure 2).

### Intraoperative saphenous vein measurement

**Figure 3:**
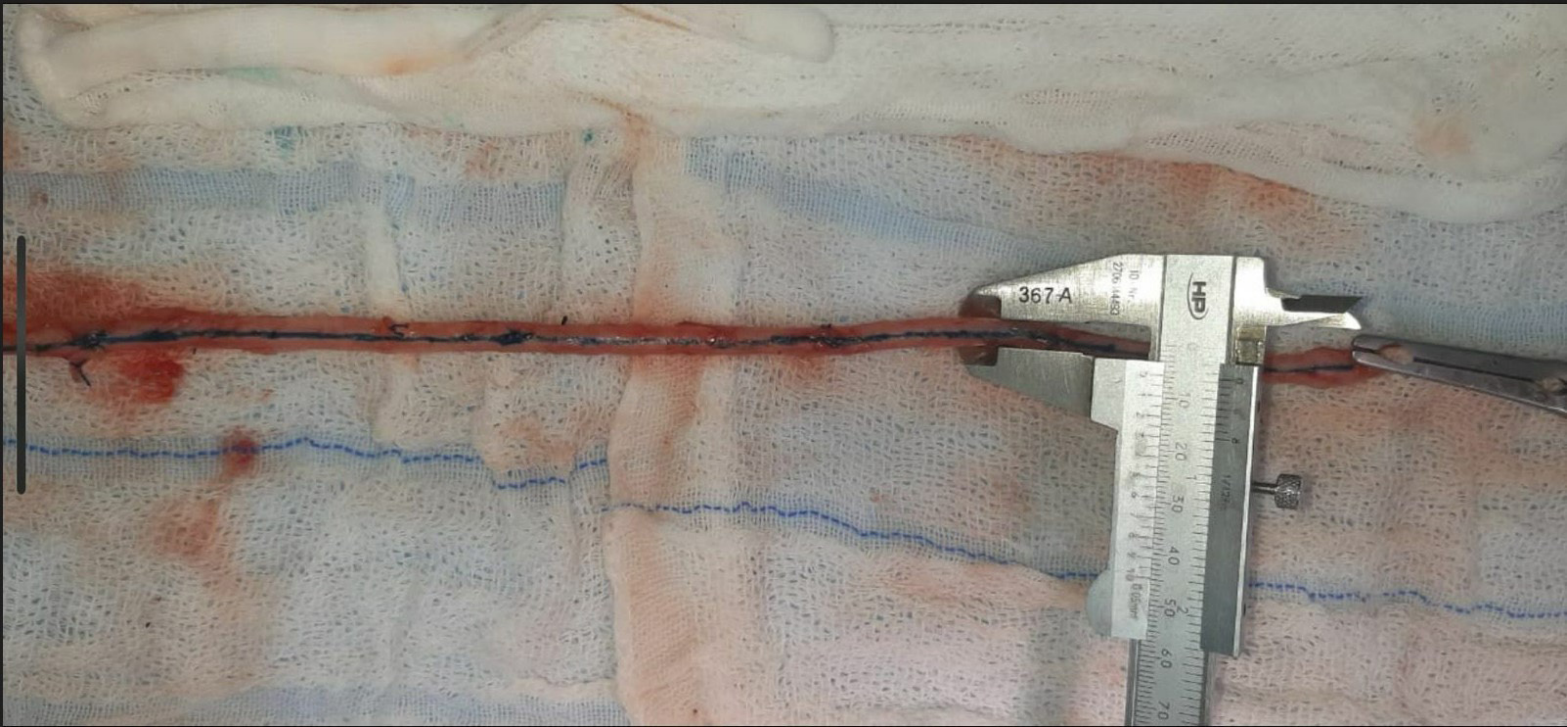
Measurement of great saphenous vein diameter during revascularization surgery using a Vernier caliper.

On the day of surgery, the surgeon responsible for preparing the GSV for use as arterial graft material duly performed hydrostatic dilatation of the saphenous vein and recorded the measurements of its external diameters with a Vernier caliper, under supervision by one of the authors (FHM) (Figure 3).

All measurements were taken at the saphenous arch (immediately after the terminal or preterminal valve, where the vein is not dilated because of the valve sinus) and then every 10 cm distally, as far as the ankle during imaging exams and intraoperatively along the entire length of the saphenous vein removed for grafting. Dilatation was achieved by proximal occlusion of the vein with a Glover atraumatic clamp and distal insertion of a number 4 urethral probe. Saline was infused via the probe with a syringe until the intravascular lumen was fully expanded, which was considered sufficient when the surgeon felt resistance to infusion of the liquid. Excessive pressure was not applied to avoid endothelial injury and minimize hyperdilatation of the vein and all measurements were supervised by the same author (FHM), to reduce the risk of bias related to vein dilatation and measurement. Demographic data on the patients and the saphenous vein measurements were recorded on a dedicated chart.

### Statistical analysis

The data collected on the charts were transferred to an electronic database (Microsoft Access 365), populating electronic spreadsheets. Data were analyzed by the statistical team, employing descriptive methods with measures of dispersion and methods for comparison of means and variance. A < 0.05 significance level was adopted.

When conducting a study, it is necessary to fit a statistical model to the data and assess them with a statistical test, adjusting the effect size to the sample size calculation. The effect size is the magnitude of the result. The greater the effect of a new intervention on the outcome, the smaller the sample size needed to prove it. Inversely, it is necessary to increase the sample size to show smaller effects. The minimum sample size was calculated for a nonparametric sample of repeated measures in one group and three data points, 5% significance, 95% power, and a 30% effect size, resulting in a necessary sample size of 39 patients. This calculation was performed using G*power version 3.1. Analysis with the F statistic was performed with the help of the statistical team at the Universidade Estadual de Campinas Medical Sciences Faculty.

## RESULTS

**Figure 4:**
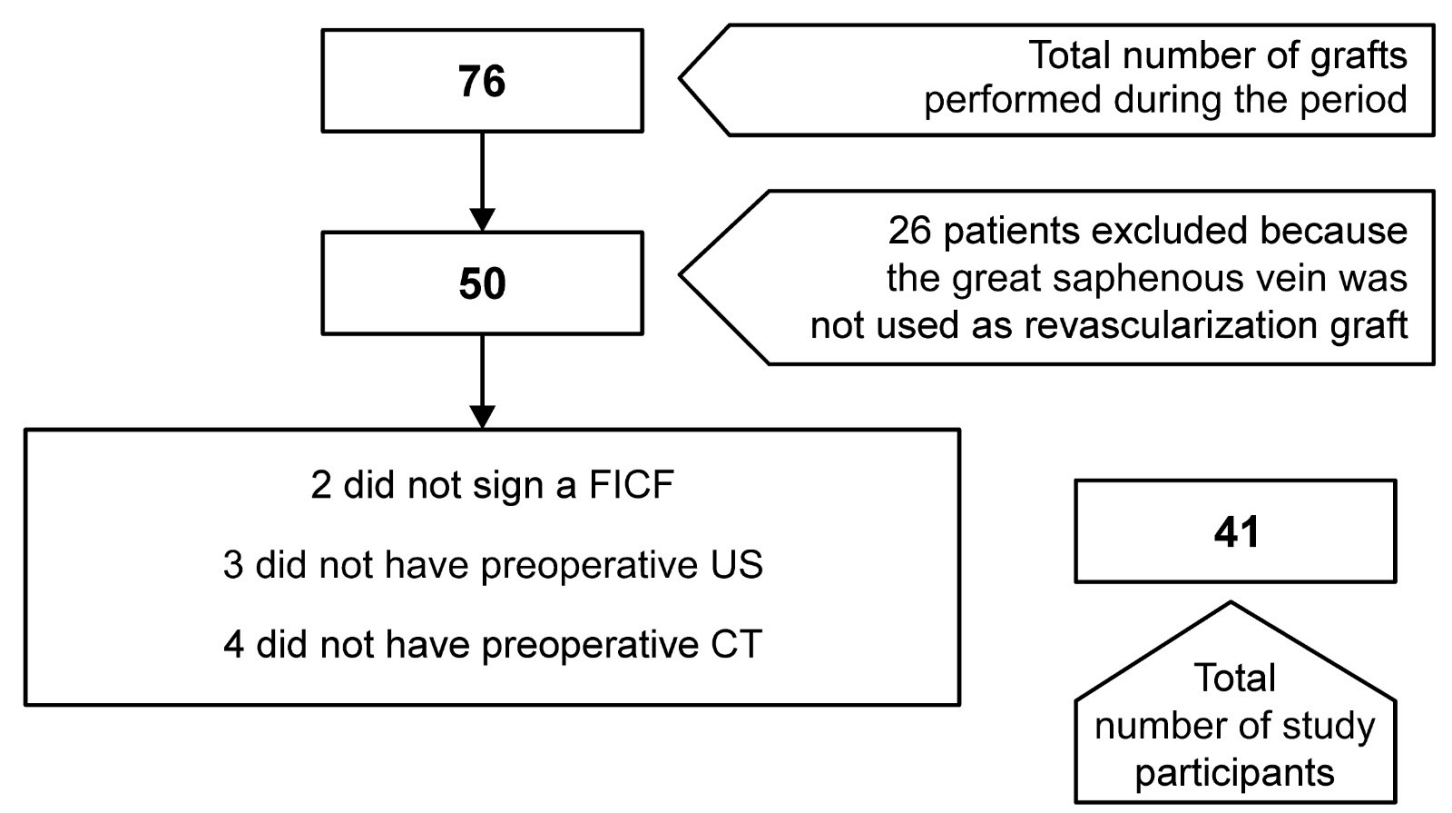
Flow diagram illustrating recruitment of patients. CT: Computed tomography; FICF: Free and informed consent form; US: Ultrasonography.

A total of 76 lower limb revascularization procedures were performed from September 2019 to December 2021. Fifty of these procedures employed the GSV as conduit. Nine patients were excluded during recruitment because they had not had preoperative US or angio-CT or because they refused to sign the consent form (Figure 4).

A total of 41 patients were treated between September 2019 and December 2021. Since the data were obtained during surgery and preoperative examinations, there was no need for follow-up and so no there were no losses after recruitment. Twenty-seven patients (65.8%) were male and mean age was 65.37 years. Smoking (current or prior) was present in 87.8% of the patients. Nineteen patients (46.3%) underwent femoropopliteal bypass and 22 (53.7%) had distal grafts. The rate of graft function was 68.3% at 30 days and 51.2% at 1 year (Table 1).

**Table 1:** Qualitative descriptive analysis of demographics.

**Variable**	**Results**
Mean age, in years (SD)	65.37 (10.91)
Male	27 (65.8%)
Chronic dialytic kidney disease	6 (14.6%)
Diabetes	21 (51.2%)
Arterial hypertension	30 (73.2%)
Smoking	36 (87.8%)
Distal graft (tibial or fibular)	22 (53.7%)
Intrahospital function rate	37 (90.2%)
30-day function rate	28 (68.3%)
1-year function rate	21 (51.2%)
BMI greater than or equal to 30 kg/m^2^	5 (12.2%)

SD: Standard deviation; BMI: Body mass index.

The saphenous vein diameters measured preoperatively were 16.4% smaller according to angio-CT and 33.8% smaller according to US, both measured in the supine position, compared to the external diameters of the saphenous veins measured intraoperatively when hydrostatically dilated. The differences between measurements during surgery (gold standard) and US and angio-CT are shown in Tables 2 and 3.

**Table 2:** Quantitative descriptive analysis of diameters measured by different methods.

	**Method**		
**Measurement**	**Computed tomography (mm) (variation from intraoperative measurement)**	**Ultrasonography (mm) (variation from intraoperative measurement)**	**Surgery (mm) (standard deviation)**
Mean	4.33 (-16.4%)	3.43 (-33.8%)	5.18 (1.23)
Arch	6.31 (-5.4%)	4.64 (-30.4%)	6.67 (1.58)
10 cm	4.44 (-17.9%)	3.60 (-33.4%)	5.41 (1.46)
20 cm	4.33 (-18.4%)	3.54 (-33.3%)	5.31 (1.46)
30 cm	4.39 (-10.9%)	3.51 (-28.8%)	4.93 (1.39)
40 cm	3.89 (-10.6%)	3.20 (-26.4%)	4.35 (1.37)
50 cm	3.52 (-12.6%)	2.73 (-32.2%)	4.03 (1.28)
60 cm	3.36 (-8.4%)	2.52 (-31.3%)	3.67 (1.50)

**Table 3:** Repeated measures ANOVA for mean caliber.

**ANOVA for repeated measures**	**p**
Mean	
Computed tomography x ultrasonography	< 0.001
Computed tomography x surgery	< 0.001
Ultrasonography x surgery	<0.001
Arch	
Computed tomography x ultrasonography	< 0.001
Computed tomography x surgery	0.1986
Ultrasonography x surgery	< 0.001
Thigh (20 cm) Computed tomography x ultrasonography	0.0001
Computed tomography x surgery	0.0005
Ultrasonography x surgery	< 0.001
Knee (30 cm)	
Computed tomography x ultrasonography	0.0001
Computed tomography x surgery	0.978
Ultrasonography x surgery	< 0.001
Leg (50 cm)	
Computed tomography x ultrasonography	0.0066
Computed tomography x surgery	0.3078
Ultrasonography x surgery	0.0013

ANOVA: Analysis of variance.

There were statistically significant differences in the means of the measurements obtained intraoperatively and with CT (p < 0.001) and in the measurements at the level of the thigh (p < 0.001), but the differences were not statistically significant for the measurements at the level of the arch (p = 0.1986), the knee (p = 0.978), or the leg (p = 0.3078) (Table 3)

Comparing the means for the intraoperative measures and the US measures, there were statistically significant differences (p < 0.05) in the measures for all levels of the lower limb. Comparison of the measurements with CT and US also revealed statistically significant differences for all measurement levels.

There were no statistical differences in GSV measurements during surgery (gold standard) when compared by patient weight and height (Table 4) or between male and female patients (Table 5).

**Table 4:** Correlation analysis of quantitative measures.

**Variable 1**	**Variable 2**	**Spearman Corr. Coef.**	**p**
Age	Mean	-0.0749	0.6460
Age	Arch	0.0495	0.7616
Age	Thigh (20 cm)	-0.1175	0.4701
Age	Leg (50 cm)	-0.0449	0.8468
			
Height	Mean	0.1895	0.2416
Height	Arch	-0.0499	0.7598
Height	Thigh (20 cm)	0.0488	0.7649
Height	Leg (50 cm)	0.1907	0.4077
			
Weight	Mean	0.0952	0.5590
Weight	Arch	0.0354	0.8282
Weight	Thigh (20 cm)	0.0300	0.8542
Weight	Leg (50 cm)	0.0440	0.8499

Spearman’s rank correlation coefficient evaluates how well the relationship between two variables can be described using a monotonic function.

**Table 5:** Comparison of great saphenous vein diameters by sex.

	**Sex**		
**Variable**	**Male**	**Female**	**p**
Mean (standard deviation)	5.13 (1.06)	5.28 (1.55)	0.8205
Arch (standard deviation)	6.37 (1.27)	7.24 (1.97)	0.1300
Thigh, 20 cm (standard deviation)	5.24 (1.20)	5.45 (1.88)	0.8974
Knee, 30 cm (standard deviation)	4.83 (1.16)	5.11 (1.78)	0.7094
Leg, 50 cm (standard deviation)	3.80 (1.24)	4.28 (1.34)	0.5009

Mann-Whitney test.

## DISCUSSION

This study was conducted while the Covid-19 pandemic was ongoing, which was a period during which normal ambulatory care for patients with peripheral arterial occlusive disease was greatly restricted, which had a negative impact on recruitment of patients for the study.

Despite the considerable advances achieved in endovascular techniques for lower limb revascularization in peripheral arterial disease, lower limb arterial bypasses remain the ideal treatment for many patients. Use of autologous vein grafts is preferred, with better long-term function rates, particularly for interventions below the knee.^2,3,9-11^

The ex vivo GSV diameter after appropriate hydrostatic dilatation is considered the gold standard, since it reveals the true elasticity and distensibility of the vein for its role as an arterial conduit, subjected to greater pressures.^7,12^ To explain the difference between the measurements observed in the preoperative examinations and the measurements performed during surgery, we can deduce that when a healthy person adopts a horizontal decubitus position, venous hydrostatic pressure reduces in the lower limbs and the diameters of the veins also reduce.^7,12^

It is important to observe that patients who have lower limb trophic ulcers find it hard to remain standing up during ultrasonographic examination and find it more comfortable and secure in horizontal decubitus. Choosing this position for the US examination also enables comparison with the CT method, which is performed in a horizontal supine position. During the US examination, it is necessary to apply a minimum of pressure to the skin, which ensures correct coupling between the transducer and the gel-coated skin, but could contribute to reducing the measurements taken with the method.

It is therefore understandable that the mean diameter measured with US was 33.8%, or 1.75 mm, smaller than the mean intraoperative measurement, since the intraoperative method was performed with the vein distended with high hydrostatic pressure. This result is similar to results in the literature.^7,12^ The GSV diameters measured with CT were slightly larger than those measured with US in our study, and a mean of 0.9 mm smaller than the intraoperative measurements, which is similar to results seen in the literature.^1^

Although CT is habitually used to assess the arterial bed, it is not routinely used to map veins.^8,13^ Assessment of saphenous vein anatomy with tomography is technically feasible and practical and CT appears to be very precise,^1,8,13^ even for assessment of venous anatomy in segments with smaller diameters.^8,13^ CT can provide data on the overall anatomy of the GSV and when it shows a visibly adequate diameter, the CT scan can be used as the only imaging exam for assessment of both arterial and venous anatomy, reducing costs.^8,13^ One exception is that confirmation with US venous mapping could be necessary if CT cannot identify an adequate vessel, which may happen if the contrast phase does not reveal the superficial venous bed.^13^

In addition to the choice of graft, preoperative assessment of patients scheduled for bypass with venous grafts for infrainguinal disease also includes assessment of the patient, of comorbidities, of demographic differences, and of the anatomy of the disease.^5^ It was assumed that female patients and those with shorter stature would have smaller saphenous diameters; but, on the contrary, comparing the vein diameters on the basis of sex, weight, height, and age variables did not reveal statistical difference between measurements. The literature is contradictory with regard to assessments of this comparison.^6,14^

## CONCLUSIONS

The caliber of the saphenous vein was underestimated by preoperative US and CT examinations in relation to the measurements recorded during surgery (considered the gold standard), with measurements that were 33.8% and 16.4% smaller respectively. Therefore, in patients scheduled for grafts for revascularization of lower limbs, the choice of graft material should take this finding into consideration to avoid prematurely ruling out use of the saphenous vein during planning. CT can be used as the only preoperative venous mapping method, reducing costs. In the present sample, age, sex, height, and weight had no influence on GSV diameter.
