# Supplementary material for: Correlations between ultrasound, tomographic, and intraoperative measurements of the great saphenous vein used as an arterial graft
Source: J Vasc Bras. 2023 May 15;22:e20220121. doi: 10.1590/1677-5449.202201212 (PMC10275644; doi:10.1590/1677-5449.202201212)
Supplement: Translation

# Translation: Correlação das medidas ultrassonográficas, tomográficas e intraoperatórias da veia safena interna utilizada como enxerto arterial

**DOI:** 10.1590/1677-5449.202201211

**Authors:** Vinicius Adorno Gonçalves, Daniel Martins Vieira Zimmermann, Fábio Hüsemann Menezes

**Affiliations:** 1 Universidade Estadual de Campinas - UNICAMP, Faculdade de Ciências Médicas, Hospital de Clínicas, São Paulo, SP, Brasil.

**Keywords:** veia safena, doença arterial periférica, ultrassonografia, tomografia

## Abstract

**Contexto:**

A veia safena interna é a principal veia superficial do membro inferior, sendo também a mais utilizada para cirurgias de enxerto arterial para revascularização de membros inferiores. O conhecimento prévio da qualidade da veia pode orientar a mudança da estratégia terapêutica, evitando cirurgias fadadas ao insucesso. Observou- se, com frequência, a discrepância entre achados intraoperatórios e exames de imagem.

**Objetivos:**

Avaliar e comparar o calibre da veia safena interna através de dois métodos de imagem [ultrassonografia (USG) dúplex e angiotomografia computadorizada (angio TC)] e do padrão-ouro (medida no intraoperatório).

**Métodos:**

Tratou-se de estudo prospectivo observacional. Os dados coletados foram obtidos dos procedimentos médicos de rotina realizados pela equipe de Cirurgia Vascular.

**Resultados:**

Foram avaliados 41 pacientes, seguidos clinicamente por 12 meses, sendo 27 (65,8%) do sexo masculino, com média de idade de 65,37 anos. Dezenove (46,3%) pacientes foram submetidos a enxerto fêmoro-poplíteo, e 22 (53,7%) a enxertos distais. Os diâmetros da veia safena foram em média 16,4% menores na TC e 33,8% menores na USG, quando medidos em decúbito dorsal no pré-operatório, comparados ao diâmetro externo após dilatação hidrostática no intraoperatório. Não houve diferença estatística das medidas da cirurgia quando se comparou sexo, peso e altura.

**Conclusões:**

A avaliação do calibre da veia safena foi subestimada pelos exames de USG e TC pré-operatórias com o paciente em decúbito dorsal, em relação à medida intraoperatória. Em pacientes em programação de enxerto para revascularização, a escolha do conduto deve levar esse dado em consideração para que não ocorra exclusão precipitada do uso da veia safena no planejamento.

## INTRODUÇÃO

A veia safena interna (VSI), também denominada veia safena magna ou longa, é a principal veia do membro inferior. Surge medialmente ao arco venoso dorsal do pé e desemboca na veia femoral, próximo ao ligamento inguinal^1-4^ . Além da drenagem venosa do membro inferior, a VSI também desempenha importante papel cirúrgico devido ao seu uso para enxertos arteriais. Um exemplo é a cirurgia de revascularização do miocárdio^5^ . Além disso, esse grande vaso também é a principal escolha nas cirurgias de pontes vasculares no caso de doença vascular periférica em membros inferiores^6^ .

São várias as vantagens do uso da VSI para a cirurgia vascular: (1) é autógena, e sabe-se que veias autógenas continuam sendo a melhor conduta, pois apresentam a maior taxa de funcionamento a longo prazo para tratamento de doenças vasculares^5^ ; (2) é longa e de fácil manuseio e dissecção^3^ ; e (3) tem grande diâmetro e espessura da parede, possibilitando a criação de diversos enxertos. Principalmente, salienta-se o fato de que, não à toa, a VSI tem sido utilizada há mais de 50 anos como enxerto arterial^7^ .

A criação de enxertos arteriais com o uso de veias, em especial da VSI, envolve uma detalhada investigação pré-operatória. A avaliação da disponibilidade da veia e sua qualidade é de significativa importância, e deve ser realizada antes de qualquer procedimento cirúrgico. Isso pode ser feito com exame físico e exame de imagem, como mapeamentos através de ultrassonografia (USG) dúplex, angiotomografia computadorizada (angio TC) e angiorressonâcia magnética nuclear^1,5,8^ . São avaliados a qualidade e o comprimento do vaso, de tal forma que veias entre 2 e 3 mm de diâmetro devam ser exploradas (veias com mais de 3 mm são consideradas bons condutos), visando sempre vasos que sejam maleáveis e compressíveis e rejeitando aqueles que sejam pequenos, calcificados ou escleróticos^5^ . O diâmetro da VSI é associado ao funcionamento do enxerto e, portanto, é um critério importante, senão o principal, para a definição da conveniência do seu uso como substituto arterial^6^ .

Apesar de várias pesquisas correlacionarem diferentes tipos de exames de imagem entre si e com o funcionamento do enxerto arterial criado a partir da VSI, principalmente no que se refere ao diâmetro do vaso utilizado, não foram encontrados estudos que indiquem a correlação entre ambos os exames e a preparação da veia durante o procedimento cirúrgico.

## OBJETIVOS

### Objetivo principal

Avaliar o calibre da VSI através da comparação de dois métodos de imagem (USG dúplex e angio TC) e um padrão-ouro (avaliação intraoperatória).

### Objetivo secundário

Avaliar se idade, sexo, altura, peso ou índice de massa corporal (IMC) podem interferir da medida da veia safena avaliada no intraoperatório.

## MÉTODOS

Estudo prospectivo, observacional, não intervencionista, em que os dados coletados foram obtidos dos procedimentos médicos de rotina realizados pela equipe de Cirurgia Vascular, tendo sido o estudo realizado nas dependências do Departamento de Cirurgia Vascular, o qual conta com completa estrutura para a realização de exames de imagem e dos procedimentos cirúrgicos. O projeto passou pelo *checklist* do Strengthening the Reporting of Observational Studies in Epidemiology (STROBE) para estudos de coorte (disponível em: https://www.strobe-statement.org/checklists/), tendo sido analisados e validados todos os 15 itens apresentados.

Todos os pacientes assinaram termo de consentimento livre e esclarecido, tendo sido o estudo aprovado pelo comitê de ética em pesquisa em seres humanos da instituição, protocolo número 3.283.138, 25 de abril de 2019; Certificado de Apresentação de Apreciação Ética (CAAE) 10697019.1.0000.5404.

### População estudada

A população estudada foi geriátrica, tendo sido composta dos pacientes internados na enfermaria de Cirurgia Vascular submetidos a revascularização aberta do membro inferior através da utilização de um enxerto autógeno de VSI. Essa decisão foi tomada em consulta ambulatorial previamente à internação do paciente, sendo os exames de imagem (USG e angio TC) normalmente realizados antes da internação a nível ambulatorial. Foram colhidos dados de pacientes cujas cirurgias foram realizadas entre setembro de 2019 e dezembro de 2021. Visando evitar viés de seleção, dentro de uma amostra de conveniência, foram listados para inclusão os pacientes submetidos a cirurgia de revascularização eletiva de membro inferior com VSI de forma contígua durante o período estudado, e todos os dados referentes à medida da veia safena obtida por diferentes métodos foram coletados e comparados no mesmo indivíduo.

### Critérios de inclusão

Foram convidados a participar do estudo todos os pacientes internados na enfermaria de Cirurgia Vascular os quais foram submetidos a revascularização eletiva aberta do membro inferior através da utilização de um enxerto autógeno de VSI.

### Critérios de exclusão

Foram excluídos os pacientes que se negaram a participar do estudo, que não assinaram o termo de consentimento, que não foram submetidos a cirurgia de revascularização com a VSI, que não realizaram angio TC no planejamento pré-operatório, ou que deixaram de realizar o exame de USG para avaliação da VSI ou as medidas de calibre da VSI durante o intraoperatório, não completando, dessa forma, os dados essenciais ao estudo.

Foram analisadas as variáveis: idade, sexo, peso, altura, presença de doença renal crônica, diabetes, hipertensão arterial e tabagismo. Foram avaliados o local de anastomose distal do enxerto (se na artéria poplítea ou nas artérias distais da perna, como tibial anterior, tibial posterior ou fibular) e a taxa de funcionamento do enxerto no período intra-hospitalar, em 30 dias e em 1 ano. Além disso, calculou-se o IMC, e foram avaliadas as medidas de diâmetro da VSI (na angio TC, no USG e na cirurgia), medidas na crossa da veia safena e 10 cm, 20 cm, 30 cm, 40 cm, 50 cm e 60 cm abaixo da crossa.

### Avaliação por angiotomografia

Uma angio TC de aorta abdominal e artérias de membros inferiores foi realizada para a avaliação do leito arterial dos membros para programação cirúrgica utilizando o aparelho Aquilion 64, da Toshiba Medical. O exame foi realizado a pedido da equipe médica seguindo o protocolo da radiologia. As medidas da veia safena foram obtidas pelo mesmo autor (VAG), no eixo perpendicular à pele, levando em consideração o diâmetro externo da parede do vaso (Figura 1).

**Figura 1:** Medida de diâmetro da veia safena interna realizada através da angiotomografia.

### Avaliação por ultrassonografia

Os pacientes agendados para a cirurgia de revascularização foram habitualmente submetidos a avaliação do calibre da VSI pela USG. Esse exame é não invasivo e indolor, e foi realizado por um médico assistente da equipe da Cirurgia Vascular na sala de procedimentos não invasivos do laboratório vascular, ou na sala de cirurgia imediatamente antes da anestesia para o procedimento cirúrgico, com a supervisão de um dos autores (FHM) e utilizando o aparelho de USG Aplio 500 Toshiba. As medidas da veia safena são realizadas no eixo perpendicular à pele, levando em consideração o diâmetro externo da parede do vaso no ponto onde a imagem é circular, com o mínimo de pressão exercida pelo transdutor sobre a pele (Figura 2).

**Figura 2:** Medida de diâmetro da veia safena interna realizada através da ultrassonografia.

### Medida intraoperatória da veia safena

No dia da cirurgia, o médico responsável pelo preparo da VSI para ser utilizada como enxerto arterial anotou, após adequada dilatação hidrostática da veia, a medida dos diâmetros externos da veia safena com um paquímetro, com a supervisão do autor FHM (Figura 3).

**Figura 3:** Medida de diâmetro da veia safena interna realizada no intraoperatório de cirurgia de revascularização com utilização de paquímetro.

Todas as medidas foram realizadas junto à crossa da veia safena (imediatamente após a válvula terminal ou pré-terminal, onde a veia não apresenta a dilatação do seio valvar), e caudalmente a cada 10 cm, até a região do tornozelo nos exames de imagem e por todo o comprimento da veia safena, que foi retirada para ser utilizada como enxerto. A dilatação foi realizada com a oclusão proximal da veia com uma pinça atraumática Glover e distalmente pela inserção de uma sonda uretral número 4. Foi realizada infusão de soro fisiológico através de uma seringa pela sonda até completa expansão do lúmen intravascular, sendo considerada adequada após a sensação de resistência à infusão de líquido pelo cirurgião. Pressões excessivas não foram realizadas para não ocasionar lesão endotelial e para minimizar a ocorrência de hiperdilatação da veia, e as medidas foram sempre supervisionadas pelo mesmo autor (FHM), para reduzir o risco de viés relacionado à dilatação e à medida da veia. Os dados demográficos dos doentes e as medidas das veias safenas foram anotados em uma ficha específica.

### Tratamento estatístico

Os dados coletados das respectivas fichas foram transferidos para banco de dados eletrônico (Microsoft Access 365) com geração de planilhas eletrônicas. Os dados foram analisados pela equipe de estatística, aplicando-se métodos descritivos com medidas de dispersão e métodos de comparação de médias e variância conforme indicado. O nível de significância adotado foi p menor que 0,05.

Para realização de um estudo, foi necessário ajustar um modelo estatístico aos dados e então avaliá-los com um teste estatístico, adequando-se o tamanho do efeito para o cálculo amostral. O tamanho do efeito é a magnitude do resultado. Quanto maior é o efeito da nova intervenção no desfecho, menor é o tamanho amostral necessário para comprová-lo. Inversamente, para mostrar efeitos menores, é necessário aumentar o tamanho amostral. Para cálculo do tamanho amostral mínimo, considerando uma amostra não paramétrica para medidas repetidas com um grupo e três tempos, uma significância de 5%, um poder de 95% e um tamanho de efeito de 30%, seria necessário um tamanho amostral de 39 pacientes. O cálculo foi realizado pelo software G*power versão 3.1. A análise estatística foi realizada com auxílio do serviço de estatística da Faculdade de Ciências Médicas da Universidade Estadual de Campinas (UNICAMP).

## RESULTADOS

Um total de 76 procedimentos de revascularização de membro inferior foram realizados entre setembro de 2019 e dezembro de 2021. Desses, 50 procedimentos utilizaram como conduto a VSI. Nove pacientes foram excluídos no período de seleção por não possuírem USG ou angio TC pré-operatória ou por recusa em assinar o termo de consentimento (Figura 4).

**Figura 4:** Fluxograma dos pacientes incluídos no estudo. TC: Tomografia computadorizada; TCLE: Termo de consentimento livre e esclarecido; USG: Ultrassonografia.

Foram incluídos ao final 41 pacientes atendidos entre setembro de 2019 e dezembro de 2021. Como os dados obtidos foram do ato operatório e exames pré-operatórios, sem necessidade de seguimento, não foram registradas perdas após a inclusão. Vinte e sete pacientes (65,8%) eram do sexo masculino, com média de idade de 65,37 anos. Tabagismo (ativo ou prévio) estava presente em 87,8% dos pacientes. Dezenove pacientes (46,3%) foram submetidos a enxerto fêmoro-poplíteo, e 22 (53,7%) a enxertos distais. A taxa de funcionamento do enxerto foi de 68,3% em 30 dias e de 51,2% em 1 ano (Tabela 1).

**Tabela 1:** Análise descritiva demográfica qualitativa.

**Variável**	**Resultados**
Idade média, em anos (DP)	65,37 (10,91)
Sexo masculino	27 (65,8%)
Doença renal crônica dialítica	6 (14,6%)
Diabetes	21 (51,2%)
Hipertensão arterial	30 (73,2%)
Tabagismo	36 (87,8%)
Enxerto distal (tibial ou fibular)	22 (53,7%)
Taxa de funcionamento intra-hospitalar	37 (90,2%)
Taxa de funcionamento em 30 dias	28 (68,3%)
Taxa de funcionamento em 1 ano	21 (51,2%)
IMC maior ou igual a 30 kg/m^2^	5 (12,2%)

DP: Desvio padrão; IMC: Índice de massa corporal.

Os diâmetros da veia safena medidos no pré-operatório foram, em média, 16,4% menores na angio TC e 33,8% menores na USG, em decúbito dorsal, quando comparados aos diâmetros externos das safenas dilatadas hidrostaticamente no intraoperatório. As diferenças entre as medidas da cirurgia (padrão-ouro) e a USG e a angio TC estão representadas nas Tabelas 2 e 3.

**Tabela 2:** Análise descritiva quantitativa do diâmetro por diferentes métodos de mensuração.

	**Método**		
**Medida**	**Tomografia computadorizada (em mm) (variação para cirurgia)**	**Ultrassonografia (em mm) (variação para cirurgia)**	**Cirurgia (em mm) (desvio padrão)**
Média	4,33 (-16,4%)	3,43 (-33,8%)	5,18 (1,23)
Crossa	6,31 (-5,4%)	4,64 (-30,4%)	6,67 (1,58)
10 cm	4,44 (-17,9%)	3,60 (-33,4%)	5,41 (1,46)
20 cm	4,33 (-18,4%)	3,54 (-33,3%)	5,31 (1,46)
30 cm	4,39 (-10,9%)	3,51 (-28,8%)	4,93 (1,39)
40 cm	3,89 (-10,6%)	3,20 (-26,4%)	4,35 (1,37)
50 cm	3,52 (-12,6%)	2,73 (-32,2%)	4,03 (1,28)
60 cm	3,36 (-8,4%)	2,52 (-31,3%)	3,67 (1,50)

**Tabela 3:** ANOVA de medidas repetidas para a média de calibre.

**ANOVA para medidas repetidas**	**Valor p**
Média	
Tomografia computadorizada x ultrassonografia	< 0,001
Tomografia computadorizada x cirurgia	< 0,001
Ultrassonografia x cirurgia	<0,001
Crossa	
Tomografia computadorizada x ultrassonografia	< 0,001
Tomografia computadorizada x cirurgia	0,1986
Ultrassonografia x cirurgia	< 0,001
Coxa (20 cm)	
Tomografia computadorizada x ultrassonografia	0,0001
Tomografia computadorizada x cirurgia	0,0005
Ultrassonografia x cirurgia	< 0,001
Joelho (30 cm)	
Tomografia computadorizada x ultrassonografia	0,0001
Tomografia computadorizada x cirurgia	0,978
Ultrassonografia x cirurgia	< 0,001
Perna (50 cm)	
Tomografia computadorizada x ultrassonografia	0,0066
Tomografia computadorizada x cirurgia	0,3078
Ultrassonografia x cirurgia	0,0013

ANOVA: Análise de variância.

Comparando as médias obtidas no intraoperatório e pela TC, houve significância estatística na diferença média das medidas (p < 0,001) e na medida realizada na altura da coxa (p < 0,001), porém não houve significância estatística nas medidas realizadas na altura da crossa (p = 0,1986), do joelho (p = 0,978) e da perna (p = 0,3078) (Tabela 3)

Comparando as médias obtidas no intraoperatório e pela USG, houve significância estatística (p < 0,05) na diferença de medidas em todos os níveis do membro inferior. A comparação entre as medidas encontradas na TC e na USG também revelou significância estatística em todos os níveis de medida.

Não houve diferença estatística das medidas da VSI na cirurgia (padrão-ouro) quando se levou em consideração o peso e a altura dos pacientes (Tabela 4), ou comparativamente entre os sexos masculino e feminino (Tabela 5).

**Tabela 4:** Análise de correlação de medidas quantitativas.

**Variável 1**	**Variável 2**	**Coef. Corr. Spearman**	**Valor p**
Idade	Média	-0,0749	0,6460
Idade	Crossa	0,0495	0,7616
Idade	Coxa (20 cm)	-0,1175	0,4701
Idade	Perna (50 cm)	-0,0449	0,8468
Altura	Média	0,1895	0,2416
Altura	Crossa	-0,0499	0,7598
Altura	Coxa (20 cm)	0,0488	0,7649
Altura	Perna (50 cm)	0,1907	0,4077
Peso	Média	0,0952	0,5590
Peso	Crossa	0,0354	0,8282
Peso	Coxa (20 cm)	0,0300	0,8542
Peso	Perna (50 cm)	0,0440	0,8499

O coeficiente de correlação de postos de Spearman avalia com que intensidade a relação entre duas variáveis pode ser descrita pelo uso de uma função monótona.

**Tabela 5:** Comparação de medidas de diâmetro da veia safena interna por sexo.

	**Sexo**		
**Variável**	**Masculino**	**Feminino**	**Valor p**
Média (desvio padrão)	5,13 (1,06)	5,28 (1,55)	0,8205
Crossa (desvio padrão)	6,37 (1,27)	7,24 (1,97)	0,1300
Coxa, 20 cm (desvio padrão)	5,24 (1,20)	5,45 (1,88)	0,8974
Joelho, 30 cm (desvio padrão)	4,83 (1,16)	5,11 (1,78)	0,7094
Perna, 50 cm (desvio padrão)	3,80 (1,24)	4,28 (1,34)	0,5009

Teste de Mann-Whitney.

## DISCUSSÃO

Este estudo foi desenvolvido durante o período de pandemia da COVID-19, período em que houve grande limitação ao atendimento ambulatorial regular de pacientes com doença arterial obstrutiva periférica, prejudicando a inclusão de pacientes no estudo.

Apesar dos avanços consideráveis ​​nas técnicas endovasculares para revascularização do membro inferior na doença arterial periférica, as pontes arteriais dos membros inferiores permanecem a terapia ideal para muitos pacientes. O uso da veia autóloga é o preferido, com melhor taxa de funcionamento a longo prazo, principalmente para intervenções abaixo do joelho^2,3,9-11^ .

O diâmetro da VSI *ex vivo*, após dilatação hidrostática adequada, é considerado o padrão-ouro, por representar a real elasticidade e distensibilidade da veia para sua atuação como conduto arterial, submetida a maiores pressões^7,12^ . Para justificar a diferença entre as medidas encontradas nos exames pré-operatórios e a medida obtida durante a cirurgia, podemos deduzir que, quando um indivíduo saudável adota a posição em decúbito horizontal, a pressão hidrostática venosa nos membros inferiores diminui, e os diâmetros das veias também diminuem^7,12^ .

Importante observar que os pacientes que apresentam lesões tróficas de membros inferiores apresentam dificuldade para manterem-se em pé durante a avaliação ultrassonográfica, sendo mais confortável e segura a realização do exame em decúbito horizontal. A opção por essa posição durante a USG também permitiu a comparação com o método de TC, que é realizado em decúbito dorsal horizontal. Durante o exame de USG é necessário um mínimo de pressão sobre a pele, para garantir o correto acoplamento do transdutor à pele com o gel, o que poderia contribuir para diminuir as medidas realizadas por esse método.

Portanto, justificou-se a média de diâmetro aferido na USG ter sido 33,8%, ou 1,75 mm, inferior à aferido no intraoperatório, uma vez que a medida intraoperatória foi realizada com a veia distendida sob alta pressão hidrostática. Esse resultado foi semelhante ao encontrado na literatura^7,12^ . Os diâmetros da VSI medidos na TC foram ligeiramente maiores do que as medidas da USG em nosso estudo, média de 0,9 mm inferior ao aferido intraoperatório, resultado semelhante ao encontrado na literatura^1^ .

Embora a TC seja habitualmente usada para avaliar o leito arterial, ela não tem sido usada rotineiramente para mapeamento de veias^8,13^ . A avaliação da anatomia venosa da veia safena pela tomografia é tecnicamente factível e prática; além disso, a TC parece ser muito precisa^1,8,13^ , inclusive para avaliação da anatomia venosa superficial em segmentos de menor diâmetro^8,13^ . A TC pode fornecer dados sobre a anatomia global da VSI e, quando esta possui diâmetro adequado visível no exame, pode ser usada como única modalidade de imagem para avaliar tanto a anatomia arterial como a venosa, reduzindo custos^8,13^ . Como exceção, a confirmação com o mapeamento venoso pela USG pode ser necessária quando a TC não identifica o conduto adequado, o que pode acontecer se o tempo de passagem do contraste não demonstrar o leito venoso superficial^13^ .

A avaliação pré-operatória de pacientes submetidos a ponte com enxerto venoso para doença infrainguinal inclui, além do conduto, a avaliação do paciente, comorbidades, diferenças demográficas e a anatomia da doença^5^ . Imaginou-se que pacientes do sexo feminino e com menor estatura apresentassem menores diâmetros de safena; no entanto, comparando-se diferentes diâmetros da veia com as variáveis sexo, peso, altura e idade, ao contrário do que poderíamos imaginar pelo senso comum, não se encontrou diferença estatística entre as medidas. A literatura é conflitante sobre a avaliação dessa comparação^6,14^ .

## CONCLUSÃO

A avaliação do calibre da veia safena foi subestimada pelos exames de USG e TC pré-operatórios em relação à medida registrada na cirurgia (considerada padrão-ouro), sendo 33,8% e 16,4% menores, respectivamente. Dessa forma, em pacientes com programação de enxerto para revascularização de membros inferiores, a escolha do conduto deve levar esse achado em consideração, para que não ocorra exclusão precipitada do uso da veia safena no planejamento. A TC pode ser utilizada como único método de mapeamento venoso pré-operatório, reduzindo custos. Nesta amostra, não houve influência da idade, sexo, altura e peso no diâmetro da VSI.
